# Human islet amyloid polypeptide aggregation upregulates the mitochondrial cholesterol transport protein StAR and induces mitochondrial dysfunction in beta cells

**DOI:** 10.1007/s00125-026-06749-8

**Published:** 2026-05-20

**Authors:** Meghan F. Hogan, Rehana Akter, Alfred C. Aplin, Andrew T. Templin, Nathalie Esser, Daniel T. Meier, Brendy-Sue Fountaine, Joseph J. Castillo, Assam El-Osta, Rebecca L. Hull-Meichle, Sakeneh Zraika, Steven E. Kahn

**Affiliations:** 1 VA Puget Sound Health Care System, Seattle, WA USA; 2 Division of Metabolism, Endocrinology and Nutrition, Department of Medicine, University of Washington, Seattle, WA USA; 3 Department of Medicine, Roudebush Veterans Affairs Medical Center, Indianapolis, IN USA; 4 Indiana University School of Medicine, Indianapolis, IN USA; 5 Laboratory of Immunometabolism and Nutrition, GIGA-I3, CHU Liège, University of Liège, Liège, Belgium; 6 Department of Biomedicine, University of Basel, Basel, Switzerland; 7 Epigenetics in Human Health and Disease Program, Baker Heart and Diabetes Institute, Melbourne, VIC Australia; 8 Department of Cell Biology, University of Alberta, Edmonton, AB Canada

**Keywords:** Amyloid, Beta cell, Cholesterol, Islet amyloid polypeptide, Mitochondrial cholesterol transport protein, Mitochondrial dysfunction, StAR, STARD1, Type 2 diabetes

## Abstract

**Aims/hypothesis:**

Loss of islet beta cell function and mass are critical in the pathogenesis of type 2 diabetes, a disease in which ~90% of individuals exhibit islet amyloid deposition. Amyloid deposits comprise the normal beta cell secretory product, human islet amyloid polypeptide (hIAPP), the aggregation of which is toxic to beta cells. While the underlying mechanism(s) for toxicity remain unknown, it is likely to involve mitochondrial dysfunction. We have shown that the mitochondrial cholesterol transport protein, steroidogenic acute regulatory protein (StAR), is upregulated in beta cells following amyloid deposition. Here, we examined the role of StAR in the toxicity of islet amyloidosis.

**Methods:**

Human islets from non-diabetic donors were cultured under amyloidogenic conditions and StAR expression was examined. StAR expression was also determined in islets isolated from transgenic mice expressing amyloidogenic hIAPP or non-transgenic littermates expressing non-amyloidogenic islet amyloid polypeptide, cultured under amyloidogenic conditions with or without the addition of an amyloid inhibitor. Total islet cholesterol content, mitochondrial cholesterol content, mitochondrial function and cell viability/death were compared in transgenic hIAPP and non-transgenic islets cultured in amyloidogenic conditions. Additionally, StAR localisation to islet cells, as well as its intracellular localisation, was examined.

**Results:**

StAR was present in human islets at the mRNA and protein level, and expression increased significantly with amyloid formation in vitro. Further, in hIAPP transgenic mouse islets, StAR expression was amyloid-dependent. StAR was predominantly expressed in beta cells, and the amyloid-induced increase in StAR protein was found specifically in the mitochondrial fraction. While total and mitochondrial cholesterol content was unchanged between non-transgenic and hIAPP transgenic mouse islets cultured under amyloidogenic conditions, increased StAR expression was associated with decreased mitochondrial glucose-stimulated respiration and increased cell death.

**Conclusions/Interpretation:**

These findings are consistent with StAR having a pathophysiological role in the beta cell in type 2 diabetes, where its upregulation under conditions of islet amyloid deposition could contribute to mitochondrial dysfunction.

**Graphical Abstract:**

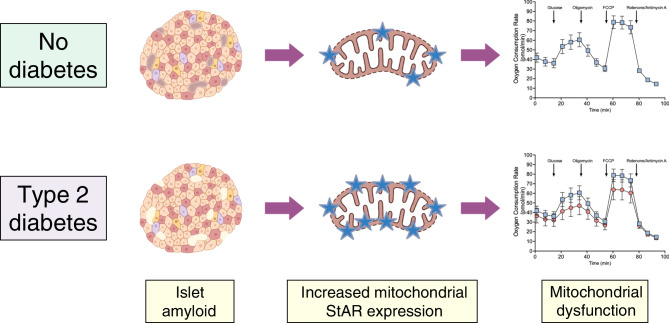

**Supplementary Information:**

The online version contains peer-reviewed but unedited supplementary material available at 10.1007/s00125-026-06749-8.


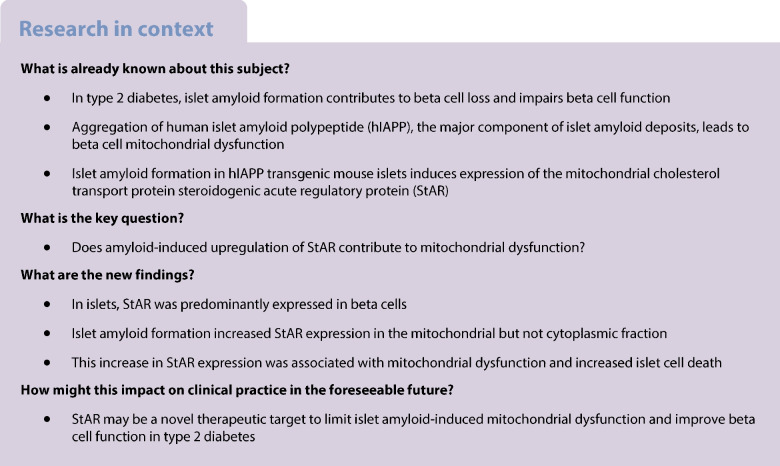


## Introduction

Pancreatic islet beta cell dysfunction and decreased beta cell mass are critical defects in the pathogenesis of type 2 diabetes, where islet amyloid is present in approximately 90% of individuals [1, 2]. Amyloid deposits contain islet amyloid polypeptide (IAPP), a normal beta cell secretory product that can aggregate, a process that is toxic to beta cells [3–5]. Studies have identified mitochondrial dysfunction as a potential mediator of IAPP-induced beta cell damage, with IAPP aggregation/islet amyloid deposition being associated with oxidative stress, cytochrome *c* release and caspase 3 activation [6, 7]. Approaches that reduce oxidative stress or suppress the mitochondrial (intrinsic) apoptotic pathway limit the toxic effects of islet amyloid deposition [6, 8]. However, how islet amyloid deposition leads to mitochondrial dysfunction has yet to be fully elucidated.

Using RNA-seq, we previously identified the gene encoding steroidogenic acute regulatory protein (StAR or STARD1) as the most highly upregulated, previously unrecognised islet amyloid-induced gene [9]. StAR is a member of the highly conserved StAR-related lipid transfer domain containing (STARD) family of lipid transport proteins [10]. In addition to StAR, another member of the STARD family of proteins, STARD3 (also known as metastatic lymph node 64 protein, MLN64), and mitochondrial translocator protein (TSPO; also previously known as peripheral benzodiazepine receptor [PBR]) act as mitochondrial cholesterol transport facilitators [11, 12]. StAR promotes cholesterol transport from the outer to the inner mitochondrial membrane, a process that comprises the rate-limiting step in mitochondrial cholesterol metabolism [13]. While StAR is important in steroidogenic tissues for the synthesis of glucocorticoids, mineralocorticoids and sex steroids [14], it is also expressed in several non-classical steroidogenic tissues, including the following: liver, where it is involved in bile acid production [15]; brain, where it enables de novo neurosteroid synthesis [16]; macrophages, where it leads to the production of oxysterol metabolites that increase transcription of ATP-binding cassette family proteins (ABCA1, ABCG1) and thereby cholesterol efflux [17]; and cardiac fibroblasts, where it increases after ischaemic injury and has an anti-apoptotic effect that helps these cells survive the stress and assist in tissue repair [18].

Given that we had previously identified StAR to be upregulated under conditions of islet amyloid formation in human IAPP (hIAPP) transgenic mouse islets [9], we studied human islets to determine whether amyloid formation similarly promotes its expression under amyloidogenic conditions. Further, given StAR’s role in facilitating mitochondrial function, we also used our hIAPP transgenic mouse model of islet amyloid formation to investigate the impact of this mitochondrial protein on mitochondrial function and cell viability.

## Methods

### Culture of human islets

Human islets from male and female non-diabetic donors were obtained from the Integrated Islet Distribution Program (IIDP; electronic supplementary material [ESM] Table 1). Islets were cultured for 14 days in RPMI 1640 medium (Lonza, Walkersville, MD, USA) containing 10% (vol./vol.) FBS (Gibco 16140071, Thermo Scientific, Waltham, MA, USA), 0.5% (vol./vol.) sodium pyruvate (Gibco 11360-070) and 0.5% (vol./vol.) penicillin/streptomycin (Gibco 15140-122), and either 5.5 or 11.1 mmol/l glucose (EMD / Millipore DX0145-1), the latter to induce amyloid deposition [19]. The studies using human islets were approved by the Veterans Affairs Puget Sound Healthcare System Institutional Review Board.

### Mouse models

Two mouse models of hIAPP expression/amyloid formation were used. In the first, mice expressing hIAPP in the endogenous mouse IAPP locus (knockin mice, produced by homologous recombination) and their wild-type mouse IAPP (mIAPP)-expressing littermates were bred on a C57BL/6NHsd background (Harlan, Indianapolis, IN, USA; RRID IMSR_ENV:HSD-044) [9, 20]. In the second, transgenic mice with hemizygous expression of hIAPP under the rat insulin promoter (RIP-hIAPP) were bred on an F1 C57BL/6 (RRID MGI:2159769) × DBA/2J background (RRID IMSR_JAX:000671) [6, 19]. Non-transgenic littermates were used as controls for both models. All animal procedures were performed with approval from the Veterans Affairs Puget Sound Healthcare System Institutional Animal Care and Use Committee and according to NIH guidelines.

### Isolation and culture of mouse Islets

Islets were isolated by collagenase digestion from pancreases of 10- to 12-week-old mice of both sexes, after which islets were cultured for up to 5 days in RPMI 1640 medium containing 10% (vol./vol.) FBS, 0.5% (vol./vol.) sodium pyruvate, 0.5% (vol./vol.) penicillin/streptomycin, and either 11.1 or 16.7 mmol/l glucose as previously described [19]. Islets from up to six mice of the same genotype were pooled and randomised into conditions in all experiments. Islets harvested and pooled on any given day were considered a single *n* value as indicated in each figure. A subset of islets was cultured for 2 days in the presence of an amyloid inhibitor, Congo Red (0.2 mmol/l; Millipore Sigma, 234610, Burlington, MA, USA) or a vehicle control (DMSO; Millipore Sigma D2650) [6]. At the end of each culture period, islets were collected for the described measurements.

### Glucose-stimulated ATP production

RIP-hIAPP transgenic and mIAPP islets were cultured in 16.7 mmol/l glucose for 4 days. Then, the islets were transferred to KRB with 2.8 mmol/l glucose for 1 h at 37°C with 5% CO_2_. Triplicate sets of ten islets from each condition were then transferred to KRB containing either 2.8 or 20 mmol/l glucose for 1 h._._ Afterwards, islet ATP content was measured using the ATPLite Luminescence Assay kit (6016943; Revvity, Waltham, MA, USA). ATP content at 20 mmol/l glucose was expressed as a fold change relative to 2.8 mmol/l glucose.

### Islet cell preparation and cell sorting

Human and mouse islets were dispersed into single cells using a non-enzymatic cell dissociation buffer (Gibco 13151014). Islet cells were sorted on an Aria II high-speed cell sorter (BD Biosciences, Franklin Lakes, NJ, USA), resulting in the separation and collection of beta cell- and non-beta cell-enriched populations as described previously [21, 22]. Islet cell fractions were harvested immediately after collection for mRNA analyses.

### Histological measurements

Amyloid prevalence and severity in human and mouse islets were determined as described [19]. Briefly, islets were fixed in 10% (vol./vol.) formalin (Millipore Sigma, HT501128), embedded in agar (BD 214050) and processed for paraffin embedding. 4–10 μm sections were incubated with an anti-insulin antibody, followed by anti-mouse Alexafluor (AF) 568-conjugated secondary antibody (ESM Table 2). Slides were counterstained with Thioflavin S (0.5% vol./vol. in water; Millipore Sigma T1892) to detect amyloid, and Hoechst 33258 (2 μg/ml; Millipore Sigma 94403) to detect nuclei. Fluorescent images of individual islets were captured on Nikon E800 or Nikon NiE epifluorescence microscopes (Nikon, Melville, NY, USA), and total islet area and Thioflavin S-positive area within each islet were measured with Nikon Elements AR software (version 5.20.01). These measurements were used to calculate amyloid prevalence (proportion of islets with amyloid) and amyloid severity as Σ thioflavin S area/Σ islet area × 100 [19, 23]. An average of 21 islets per condition per experimental replicate was scored, with the observer blinded to the experimental culture conditions of the islet samples.

For the determination of StAR localisation to mitochondria, 4 μm paraffin sections of RIP-hIAPP transgenic islets that had been cultured for 6 days in 16.7 mmol/l glucose were incubated with anti-StAR and anti-TOMM20 antibodies (ESM Table 2), followed by anti-mouse AF568 and anti-rat AF488 secondaries, respectively. Fluorescent images of individual islets were captured on a Nikon A1R confocal microscope and imported to the ImageJ ‘JACoP’ plugin [24]. Calculations for Pearson correlation coefficients (PCCs) were performed on individual islets (*n*=16) and from 3 or 4 random regions of interest (ROIs) from within each islet (*n*=55 ROIs from 16 islets).

All antibodies were either validated in-house or selected based on the literature. IgG isotype negative control staining was performed for all antibodies used for immunohistochemistry.

### RNA isolation and quantitative PCR

For quantitative PCR (qPCR), total RNA was isolated and reverse transcribed as previously described [9, 21]. mRNA levels were measured in triplicate using TaqMan Gene Expression assays (Thermo/Life Technologies, Waltham, MA, USA). The specific primer probe sets used are listed in ESM Table 3. The gene encoding cyclophilin was the endogenous housekeeping gene. Gene expression levels were calculated relative to the appropriate experimental control using the ${2}^{{-\Delta \Delta \mathrm{C}}_{\mathrm{t}}}$ method [25].

### Western blotting

Islet or cell lysates (20–40 µg total protein) were separated by Tris-glycine SDS-PAGE and transferred to PVDF membranes as described [9]. After blocking non-specific binding with 5% milk/TBST (wt/vol.), membranes were probed with primary antibodies (ESM Table 2) in 5% BSA/TBST and detected with a goat anti-rabbit horseradish peroxidase secondary antibody (1:3000; P044801-2; Agilent Technologies, Santa Clara, CA, USA) in 5% milk/TBST using enhanced chemiluminescence (SuperSignal West Femto Chemiluminescent Substrate, Thermo Fisher Scientific). Densitometry was performed using ImageJ software (version 1.54g, http://imagej.org), and protein levels were reported as the ratio of the protein of interest to the housekeeping protein and expressed relative to the appropriate experimental control condition.

### Islet subcellular fractionation

Mitochondrial and cytoplasmic fractions were collected from RIP-hIAPP transgenic mouse islets (≥300 per condition), using a cell fractionation kit (ab109719; Abcam, Cambridge, MA). The protein concentration of each fraction was quantified via the bicinchoninic acid (BCA) assay, run on a western blot, and StAR protein was expressed relative to loading controls for cytoplasmic (HSP90) or mitochondrial (ATP5a) proteins.

### Cell viability

To determine cell viability, 20–25 islets/well were incubated in a 96-well plate with CellTitre-Fluor (CTF; product G6080; Promega, Madison, WI, USA) reagent for 1 h. Cell viability was measured in duplicate using a Varioskan microplate reader at 380–400 nm excitation wavelength/505 nm emission wavelength. Data are expressed as % relative to the control condition.

### Assessment of caspase 3/7 activity and cell death in islet cultures

Cell death was quantified in real time using an IncuCyte S3 live-cell imaging and analysis instrument (Sartorius, Göttingen, Germany) as described [26]. Briefly, single intact RIP-hIAPP transgenic islets were added to each well of a 96-well round-bottom plate (7007; Corning) containing RPMI media with 10% FBS and 16.7 mmol/l glucose. Either Sytox Green (100 nmol/l; S7020; Thermo Fisher Scientific) or caspase 3/7 dye (5 μmol/l; 4440; Sartorius) was included with the media at time 0. Fluorescent and phase-contrast images of each islet were taken with a ×10 objective every 4 h for 4 days. Fluorescence intensity was normalised to islet brightfield area for each islet using the IncuCyte Spheroid Module (Sartorius). Data were collected from four sets of independently isolated islets. Caspase 3/7 activation was quantified in a total of 48–60 islets and Sytox Green cell death in a total of 51 individual islets.

### Total islet cholesterol content

Forty to 50 islets were lysed in PBS buffer containing 0.1% Triton-X. Total cholesterol content in lysates was quantified using the Amplex Red cholesterol assay kit (A12216; Thermo Fisher Scientific). Total islet cholesterol content was normalised to total protein content, measured using the BCA assay (23225; Thermo Fisher Scientific).

### Mitochondrial cholesterol content

A modification of the protocol described by Wieckowski et al [27] was used to prepare mitochondrial extracts for measuring cholesterol content. Briefly, 150–200 islets were cultured, collected, and frozen at −80°C. Thawed islets were homogenised in ice-cold sodium chloride–Tris-EDA (STE) buffer using a 22G needle and centrifuged at 1000 *g* for 10 min. The supernatant fraction was re-centrifuged at 10,400 *g* for 10 min, and the resulting pellet was suspended in ice-cold STE buffer. The suspension was then centrifuged at 16,000 *g* for 2 min, and PBS buffer containing 0.1% Triton-X was added. Mitochondrial cholesterol content was measured using the Amplex Red cholesterol assay, with normalisation to mitochondrial protein content as described above for total cholesterol content.

### Mitochondrial oxygen consumption rate

To assess mitochondrial function, 20 islets/well were plated into a spheroid microplate (102978-100; Agilent Technologies) containing RPMI growth medium (103576-100; Agilent Technologies) with 2.8 mmol/l glucose. Prior to running the assay, islets were incubated in the absence of CO_2_ at 37°C for 60 min. Microplates with islets were placed into a Seahorse XF96 Analyzer (Agilent Technologies) to measure the basal mitochondrial oxygen consumption rate (OCR), followed by sequential OCR measurements after addition of 20 mmol/l glucose (stimulated), 5 μmol/l oligomycin (inhibited), 2 μmol/l carbonyl cyanide 4-(trifluoromethoxy) phenylhydrazone (FCCP; maximal) and then 3 μmol/l rotenone and antimycin (non-mitochondrial).

### Statistics

Data are presented as mean ± SEM. Significant differences between the two experimental groups were determined using a Student's *t* test. For differences between more than two groups, mean data were compared by one- or two-way ANOVA with a Holms–Sidak post hoc test to correct for multiple comparisons. Correlations were performed using PCC and Spearman's rank correlation. A *p* value of ≤0.05 was considered statistically significant. Prism (version 10.1; GraphPad, San Diego, CA, USA) was used for statistical analysis.

## Results

### StAR is expressed in human islets and increases with amyloid deposition

**Fig. 1:**
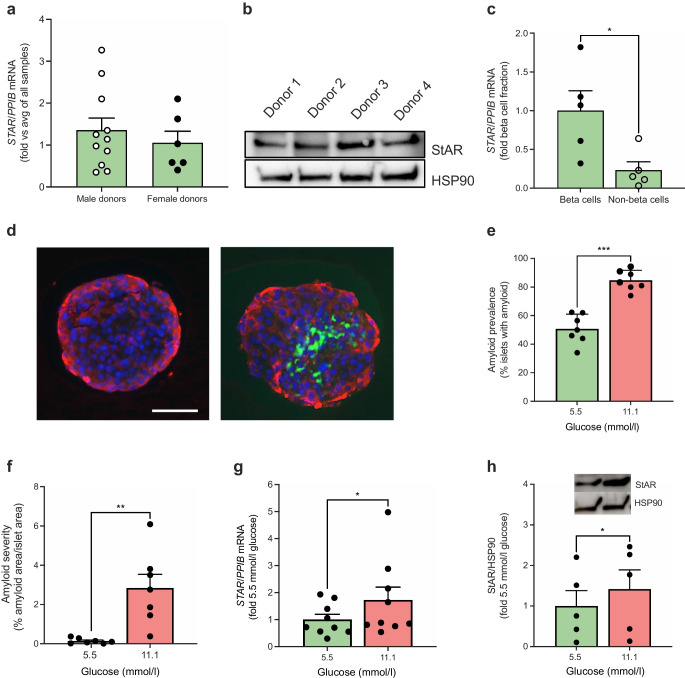
StAR is expressed in human islets and abundance increases with amyloid deposition. (**a**) *STAR* mRNA expression in islets from male (white circles; *n*=11 donors) and female (filled circles; *n*=6 donors) donors. (**b**) Representative western blots of StAR and HSP90 protein levels in islets from male and female donors. (**c**) *STAR* mRNA expression in beta (black circles) and non-beta cell (white circles) fractions isolated from human islets (*p<0.05, *n*=5 donors). (**d**) Representative images of human islets cultured for 14 days at low (5.5 mmol/l, left panel) and high (11.1 mmol/l, right panel) glucose, stained for insulin (red), amyloid (green) and nuclei (blue). Scale bar, 100 μm. (**e**, **f**) Amyloid prevalence (% of islets with amyloid) (**e**) and amyloid severity (% amyloid area/islet area) (**f**) for isolated islets cultured in 5.5 mmol/l and 11.1 mmol/l glucose (**p<0.01, ***p<0.001, *n*=7 donors). (**g**) Relative *STAR* mRNA expression in human islets cultured for 14 days at 5.5 or 11.1 mmol/l glucose (*p<0.05, *n*=9 donors). (**h**) StAR protein expression in human islets cultured for 14 days (*p<0.05, *n*=5 donors), with representative blot images shown. Avg, average

We first examined whether StAR is expressed in islets isolated from human donors (ESM Table 1). Expression of the *STAR* gene was detectable in human islets from both male and female donors with no difference between the sexes (Fig. 1a). StAR protein was also expressed in islets (Fig. 1b, h). *STAR* mRNA expression was observed in all islet preparations, with expression being predominantly in beta cells (Fig. 1c). While islet StAR expression varied between individuals, there was no relationship between StAR expression and age (*r*=−0.0136, *p*=0.50), BMI (*r*=−0.2178, *p*=0.45) or HbA_1c_ (*r*=0.2488, *p*=0.33) (ESM Fig. 1).

To determine whether StAR expression increases with amyloid formation, human islets from non-diabetic donors were cultured for 14 days in 5.5 or 11.1 mmol/l glucose (considered ‘normal’ or ‘high’ glucose, respectively). A glucose concentration of 11.1 mmol/l has previously been shown to induce islet amyloid formation in cultured human islets [28]. The prevalence of islet amyloid increased when islets were cultured in high glucose (Fig. 1d, e). Further, the severity of amyloid deposition increased in islets after culture at 11.1 mmol/l glucose (Fig. 1f). Following 14 days of culture in 11.1 mmol/l glucose, both *STAR* gene (Fig. 1g, *p*<0.05) and StAR protein (Fig. 1h, *p*<0.05) expression increased compared with that in 5.5 mmol/l glucose. These results are similar to those we reported previously with RIP-hIAPP transgenic mouse islets cultured under amyloid-forming conditions [9].

### Islet amyloid formation is associated with increased StAR expression over time

**Fig. 2:**
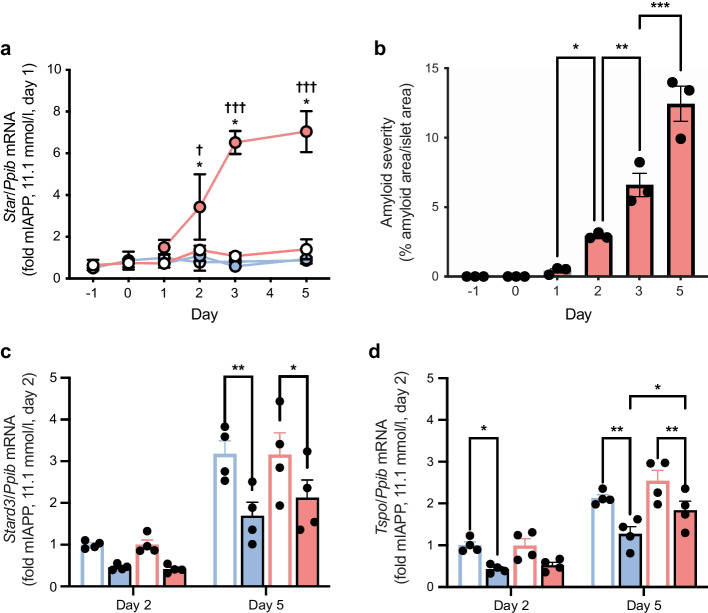
Islet *Star* expression increases with amyloid formation. (**a**) Time course of *Star* mRNA expression relative to the housekeeping gene *Ppib* in non-transgenic (mIAPP; blue lines; *n*=3 or 4) or knockin hIAPP (transgenic; red lines; *n*=3–5) mouse islets cultured in 11.1 mmol/l glucose (white circles) or 16.7 mmol/l glucose (blue circles and red circles) for the indicated number of days (*p<0.05 vs mIAPP islets at 16.7 mmol/l glucose; ^†^*p*<0.05 vs hIAPP transgenic islets at 11.1 mmol/l glucose; ^†††^*p*<0.001 vs hIAPP transgenic islets at 11.1 mmol/l glucose, *n*=3–5). (**b**) Time course of amyloid severity (% amyloid area/islet area) of knockin hIAPP islets cultured in 16.7 mmol/l glucose (*p<0.05, **p<0.01, ***p<0.001, *n*=3). (**c**, **d**) Quantification of *Stard3* (**c**) or *Tspo* mRNA (**d**) in non-transgenic (mIAPP; blue bars) and hIAPP transgenic (red bars) mouse islets cultured for 2 or 5 days in 11.1 (open bars) or 16.7 mmol/l (solid bars) glucose (*p<0.05 and **p<0.01, *n*=4)

We next determined the time course of islet *Star* gene expression relative to that of amyloid deposition in mouse models. As hIAPP but not mIAPP is amyloidogenic, we used islets from mouse models that express hIAPP, along with islets from non-transgenic control mice [20, 29]. Islets were isolated and cultured in low (11.1 mmol/l, non-amyloidogenic) and high (16.7 mmol/l, amyloidogenic) glucose conditions [19]. In hIAPP transgenic mouse islets cultured at 16.7 mmol/l glucose, *Star* gene expression progressively increased over 5 days (Fig. 2a) in a pattern similar to that observed for amyloid deposition (Fig. 2b). Further, *Star* expression in hIAPP transgenic islets cultured at 16.7 mmol/l glucose was significantly higher after 2 days of culture when compared with hIAPP transgenic mouse islets cultured at 11.1 mmol/l glucose (non-amyloid-forming conditions) or non-transgenic mouse islets cultured at 16.7 mmol/l glucose. Expression remained significantly higher throughout the rest of the culture period.

We next examined whether STARD3 and TSPO were altered by glucose or amyloid to determine whether other cholesterol transport genes are regulated similarly to StAR. We quantified mRNA expression in both hIAPP transgenic and non-transgenic mouse islets cultured for 2 or 5 days in 11.1 mmol/l or 16.7 mmol/l glucose. Unlike *Star*, the expression of both *Stard3* and *Tspo* decreased in hIAPP transgenic and non-transgenic mouse islets when cultured in high glucose vs low glucose (Fig. 2c, d).

### Increased StAR expression occurs downstream of amyloid formation

**Fig. 3:**
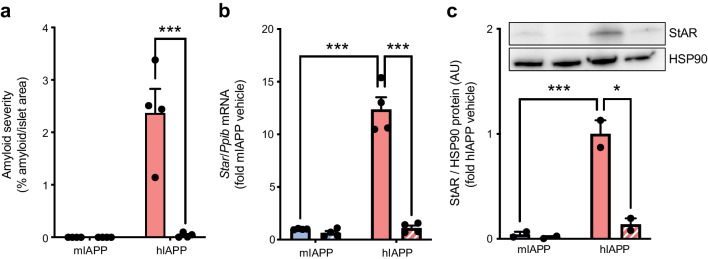
Upregulation of StAR expression occurs downstream of islet amyloid formation. Amyloid severity (**a**), *Star* mRNA expression (**b**) and StAR protein expression (**c**) in non-transgenic (mIAPP; blue bars) and RIP-hIAPP transgenic (red bars) mouse islets cultured in 16.7 mmol/l glucose with 200 μmol/l Congo Red (hatched bars) or vehicle (filled bars) for 2 days, with the representative immunoblot image shown (*p<0.05, ***p<0.001, *n*=2–4). AU, arbitrary units

To determine whether the increase in StAR expression was dependent on the formation of amyloid per se, RIP-hIAPP transgenic mouse islets were cultured for 2 days in the presence or absence of the amyloid inhibitor Congo Red [6]. Congo Red treatment prevented the increase in amyloid deposition (Fig. 3a) and similarly prevented amyloid-induced upregulation of StAR gene and protein expression (Fig. 3b, c). No change in StAR expression was observed in non-transgenic islets cultured with or without Congo Red (Fig. 3b, c).

### StAR is localised predominantly in beta cell mitochondria

**Fig. 4:**
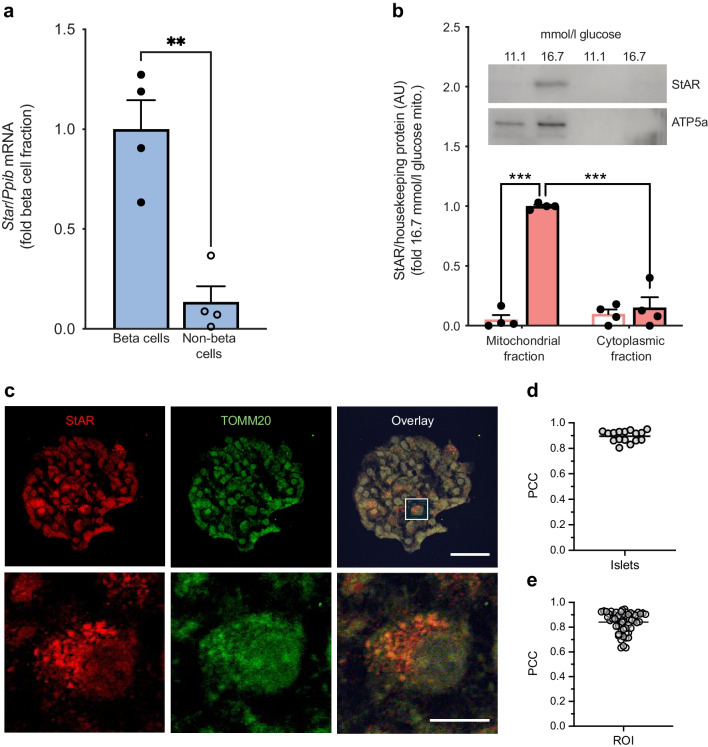
StAR is expressed predominantly in the mitochondria of beta cells and its levels of expression are increased in the mitochondrial fraction of RIP-hIAPP transgenic mouse islets under amyloid-forming conditions. (**a**) *Star* mRNA is detected at a 7.5-fold higher level in the beta cell fraction of sorted islet cells vs non-beta cells (**p<0.01, *n*=4). (**b**) StAR protein expression in mitochondrial and cytoplasmic fractions in RIP-hIAPP transgenic mouse islets cultured in 11.1 mmol/l (white bars) or 16.7 mmol/l glucose (red bars) for 7 days, with representative blot images shown (***p<0.001, *n*=4). (**c**) Co-localisation of StAR with the mitochondrial marker TOMM20. Confocal images of a RIP-hIAPP transgenic mouse islet cultured for 7 days in 16.7 mmol/l glucose stained for StAR (red) and TOMM20 (green), with channel overlay, are shown (scale bar, 50 µm) together with images of a selected ROI (lower panels; scale bar, 100 µm) indicated by the white box. (**d**, **e**) Graphs indicating the range of calculated PCCs from islets (**d**; PCC=0.896, *n*=16) and from individual ROIs (**e**; PCC=0.839, *n*=55). AU, arbitrary units; mito, mitochondrial fraction

Next, we investigated StAR's localisation within the islet. *Star* mRNA was measured in FACS-enriched beta cell and non-beta cell fractions from C57BL/6J mouse islets [22]. As we observed with human islets, *Star* gene expression was highest in the beta cell-enriched fraction (Fig. 4a), being 7.5-fold higher than in the non-beta cell fraction. StAR protein was detected exclusively in the mitochondrial fraction of RIP-hIAPP transgenic mouse islets, and was upregulated by more than tenfold following culture in 16.7 mmol/l glucose (Fig. 4b).

To determine the cellular localisation of StAR within islets, we performed double immunofluorescence confocal microscopy with StAR and the mitochondrial outer membrane protein TOMM20 (Fig. 4c). Image analysis of whole islets revealed that StAR and TOMM20 proteins were co-localised within the islet, with a mean PCC of 0.896±0.011 (Fig. 4d). The selected ROIs from within each StAR/TOMM20 co-stained islet had a mean PCC of 0.839±0.011, again indicating a high degree of correlation between the expression of these two proteins (Fig. 4e) and supporting the conclusion that StAR is expressed within mitochondria of beta cells.

### Increased StAR expression in hIAPP transgenic mouse islets is associated with mitochondrial dysfunction

**Fig. 5:**
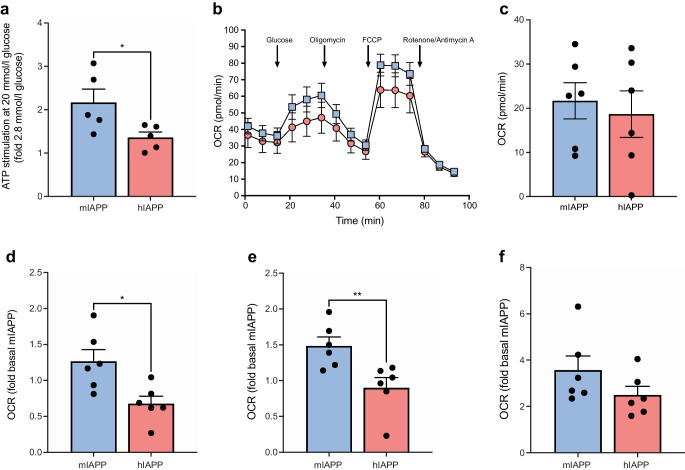
Islet amyloid-induced StAR upregulation is associated with mitochondrial dysfunction. Non-transgenic (mIAPP; blue bars) and RIP-hIAPP transgenic (red bars) mouse islets were cultured at 16.7 mmol/l glucose for 5 days prior to analysis. (**a**) ATP stimulation after treatment with 20 mmol/l glucose (*p<0.05, *n*=5). (**b**) Graph of OCR over 96 min, indicating the mitochondrial respiration profile of mIAPP islets (blue squares) and RIP-hIAPP islets (red circles) undergoing treatment with respiratory-stimulating or inhibiting compounds at the indicated timepoints. (**c**) Basal OCR in islets cultured in 2.8 mmol/l glucose. (**d**) Mitochondrial respiration after the addition of 20 mmol/l glucose. (**e**) Mitochondrial ATP-coupled respiration measured after addition of 5 µmol/l oligomycin. (**f**) Maximal mitochondrial respiration after the addition of 2 µmol/l FCCP. In all graphs *p<0.05 and **p<0.01; *n*=5 for panel **a**, *n*=6 for panels **b**–**f**

We next analysed the effect of amyloid-induced StAR expression on ATP production and mitochondrial function in mouse islets cultured for 5 days in 16.7 mmol/l glucose. Upon stimulation with 20 mmol/l glucose, ATP production was decreased by 37% in hIAPP transgenic mouse islets compared with non-transgenic islets (Fig. 5a). Additionally, we quantified mitochondrial OCR with the Seahorse Mito Stress test (Fig. 5b). Amyloid formation in hIAPP transgenic islets did not significantly change basal OCR at 2.8 mmol/l glucose (Fig. 5c). However, there was a 46% reduction in glucose-stimulated OCR (Fig. 5d, *p*<0.05), a 39% decrease in ATP-coupled OCR (Fig. 5e, *p*<0.01) and a 30% reduction in maximal OCR (Fig. 5f, *p*=0.0931) in RIP-hIAPP transgenic mouse islets, compared with non-transgenic islets.

### Islet amyloid-induced StAR expression in hIAPP transgenic mouse islets is associated with decreased cell viability and increased cell death

To investigate whether the amyloid-induced StAR expression increases total islet or mitochondrial cholesterol content, RIP-hIAPP transgenic or non-transgenic mouse islets were cultured at 11.1 or 16.7 mmol/l glucose for 120 h. Amyloid formation did not change total islet or mitochondrial cholesterol content of RIP-hIAPP transgenic compared with non-transgenic islets (ESM Fig. 2).

**Fig. 6:**
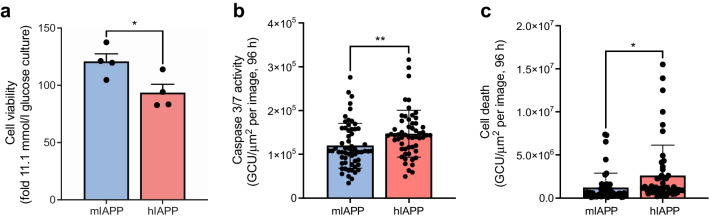
Islet amyloid-induced StAR upregulation is associated with decreased cell viability. Non-transgenic (mIAPP; blue bar) and RIP-hIAPP transgenic (red bar) mouse islets were cultured at 16.7 mmol/l glucose for 5 days. (**a**) Cell viability as measured by CTF assay (*p<0.05, *n*=4). (**b**, **c**) Caspase 3/7 activity (**b**; **p<0.01, *n*=48–60) and Sytox fluorescence (**c**; *p<0.05, *n*=51) quantified in non-transgenic and RIP-hIAPP transgenic mouse islets cultured at 16.7 mmol/l glucose for 4 days. GCU, green calibrated units (GCU/µm^2^ per image is a measure of fluorescence over the image area)

Amyloid formation in RIP-hIAPP transgenic mouse islets was associated with a 22% reduction in cell viability, when compared with non-transgenic islets (Fig. 6a). To further examine the effect of amyloid-induced StAR expression on cell death in islets, we analysed caspase 3/7 activation and Sytox Green uptake in live cultures of islets. Compared with non-transgenic islets, hIAPP transgenic islets exhibited an 18% increase in caspase 3/7 activation (Fig. 6b) and a 53% increase in Sytox Green fluorescence (Fig. 6c) after 96 h culture in 16.7 mmol/l glucose.

## Discussion

In the present study, we confirmed the presence of the cholesterol transport protein StAR in the pancreas [30], further demonstrating that it is expressed in beta cells within human islets. As we previously showed in hIAPP transgenic mouse islets [9], StAR expression in human islets is upregulated under conditions of amyloid deposition. Further, we found in both human and mouse islets that the gene encoding StAR is expressed almost exclusively in beta cells and is predominantly localised to mitochondria, where StAR protein levels are increased by islet amyloid deposition. This effect appears to be specific to StAR, as expression in mice of two other major cholesterol transport genes *Stard3 and Tspo* is decreased, rather than increased, under amyloid-forming conditions. Additionally, upregulation of StAR expression in islets is not observed upon co-culture with the amyloid inhibitor dye Congo Red, consistent with StAR induction occurring downstream of amyloid deposition.

IAPP aggregation contributes to islet dysfunction and loss of beta cell mass in type 2 diabetes [1, 2, 29]. We and others have reported that amyloid deposition is associated with mitochondrial dysfunction [6, 31]. The current study extends those observations and shows that the amyloid-induced increase in beta cell StAR expression is associated with mitochondrial dysfunction, manifest as reduced mitochondrial glucose-stimulated respiration and ATP-coupled respiration. This effect of StAR is in keeping with our previous findings that StAR overexpression in INS-1 cells results in mitochondrial dysfunction [32]. Further, we found that increased StAR expression is associated with decreased cell viability and cell death. Collectively, these findings suggest that the formation of amyloid deposits within pancreatic islets induces upregulation of StAR, which subsequently results in mitochondrial dysfunction within beta cells. Thus, it could be envisaged that islet amyloid deposition over the lifetime of people with type 2 diabetes could chronically increase StAR expression in beta cells, leading to mitochondrial dysfunction and, subsequently, beta cell dysfunction and death [33].

While, to our knowledge, no other studies have investigated StAR in the islet, there exists a prior description of StAR protein in pancreatic extracts from lean and obese male Zucker (*fa/fa*) rats [34]. Consistent with our data from non-transgenic islets cultured at high glucose, findings from the Zucker rat show no difference in StAR expression in pancreases of obese animals compared with lean controls, despite them having a 3.2 mmol/l increase in plasma glucose [34]. However, in these obese rats that exhibited mild dysglycaemia, dyslipidaemia and hyperinsulinaemia, TSPO expression was upregulated. This contrasts with our observation of a reduction in *Tspo* in non-transgenic and hIAPP transgenic mouse islets cultured at elevated glucose. These findings suggest that dyslipidaemia in obese animals may regulate islet TSPO expression differently than hyperglycaemia alone.

Could StAR have a physiological role in the islet independent of amyloid formation? First, several studies have suggested that StAR upregulation in non-steroidogenic tissues may be beneficial. In high-fat-fed mice with fatty liver, StAR overexpression regulated the expression of genes involved in hepatic lipid/glucose metabolism via bile-acid-mediated activation of the FXR–SHP pathway and improved whole-body insulin sensitivity and glucose homeostasis [35]. In the brain, StAR is developmentally regulated and its expression is elicited as a neuroprotective response to brain injury [36], including under the amyloidogenic conditions of Alzheimer's disease [37]. In the heart post-myocardial infarction, StAR is upregulated in cardiac fibroblasts, having an anti-apoptotic effect and thus allowing the cells to survive and participate in tissue repair [18]. Second, in addition to steroid production, cholesterol transport into mitochondria is important for the synthesis of bile acids in several tissues [15, 38]. The production of bile acids from cholesterol in mitochondria can be initiated by the action of the side-chain cleavage enzyme CYP27A1 which generates the oxysterol, 27-hydroxycholesterol (27-HC [17]). As human beta cells express CYP27A1 [39], 27-HC could conceivably be further metabolised into bile acids [40] or may act as a ligand for the liver X receptor (LXR), activation of which results in improved insulin release [41, 42]. Third, it is possible that StAR’s role in the islet under physiological conditions is important for the beta cell’s normal secretory function, although increasing StAR expression may result in mitochondrial dysfunction and secretory dysfunction. Further investigation using islets lacking StAR will be needed to determine whether StAR plays a role under normal conditions.

Increased islet cholesterol content has been associated with beta cell mitochondrial dysfunction, secretory dysfunction and increased blood glucose in vivo [43–46]. In line with this, we previously observed that exposure of islets to increased exogenous cholesterol resulted in increased StAR expression, increased mitochondrial cholesterol accumulation and mitochondrial dysfunction [33]. In the present study, along with the amyloid-induced increase in StAR expression, we also observed mitochondrial dysfunction; however, this was independent of any change in mitochondrial cholesterol content. Since islets in the present study were not exposed to increased exogenous cholesterol, it is possible that the cellular cholesterol levels were not sufficiently elevated to observe measurable changes in mitochondrial cholesterol upon StAR upregulation. The mechanism(s) by which amyloid-induced increases in StAR expression alter the function of beta cells requires further study. One possibility could involve disruption of mitochondrial membrane integrity due to the increased presence of StAR, which could lead to depletion of mitochondrial glutathione. While the latter typically occurs with increased cholesterol [47], increased expression of StAR in the mitochondrial membrane could disrupt the shuttling of glutathione into mitochondria by the 2-oxoglutarate (OGC; SLC25A11) and dicarboxylate (DIC; SLC25A10) carriers [47, 48]. Glutathione depletion would result in mitochondrial dysfunction, increased reactive oxygen species (ROS) production, oxidation of membrane lipids such as cardiolipin, and apoptosis [47]. In addition to the mitochondrial dysfunction observed in this study, this would be in keeping with our previous finding that islet amyloid formation induces oxidative stress whereas blocking oxidative stress reduces amyloid formation and beta cell loss [6].

In summary, we have demonstrated upregulation of the cholesterol transport protein StAR in islets and specifically within the mitochondria of beta cells, under conditions of amyloid formation. Increased expression of StAR results in decreased islet mitochondrial function and a decline in cell viability. As islet amyloid is a prevalent feature of human type 2 diabetes, and we were able to confirm that IAPP aggregation leads to an increase in StAR expression in isolated human islets, we believe our observations provide the impetus for further studies to better understand the role of islet StAR in human health and type 2 diabetes.

## Supplementary Information

Below is the link to the electronic supplementary material.

ESM (PDF 366 KB)

## Data Availability

The data that support the findings of this study are available from the corresponding author upon reasonable request.

## Abbreviations

AF: Alexafluor
BCA: Bicinchoninic acid
CTF: CellTitre-Fluor
FCCP: 4-(Trifluoromethoxy) phenylhydrazone
27-HC: 27-Hydroxycholesterol
hIAPP: Human IAPP
IAPP: Islet amyloid polypeptide
mIAPP: Mouse islet amyloid polypeptide
OCR: Oxygen consumption rate
PCC: Pearson correlation coefficient
RIP: Rat insulin promoter
ROI: Region of interest
StAR/STARD1: Steroidogenic acute regulatory protein
STARD: StAR-related lipid transfer domain containing
STE: Sodium chloride–Tris-EDA
TSPO: Translocator protein
