# Supplementary material for: Human islet amyloid polypeptide aggregation upregulates the mitochondrial cholesterol transport protein StAR and induces mitochondrial dysfunction in beta cells
Source: Diabetologia. 2026 May 20;69(8):2294–306. doi: 10.1007/s00125-026-06749-8 (PMC13310199; doi:10.1007/s00125-026-06749-8)
Supplement: Supplementary file 1 — ESM (PDF 366 KB) [file 125_2026_6749_MOESM1_ESM.pdf]

ESM (Electronic Supplementary Material)

Human Islet Amyloid Polypeptide Aggregation Upregulates the Mitochondrial Cholesterol Transport Protein, StAR, and Induces Mitochondrial Dysfunction in Beta Cells

Title	Description
ESM Table 1	Human islet donor characteristics
ESM Table 2	Primary and secondary antibodies and buffers for protein detection by western blotting, immunohistochemistry, or immunofluorescence
ESM Table 3	TaqMan probes for gene expression analysis
ESM Figure 1	Relationship of StAR mRNA expression in human islets with age, BMI and haemoglobin A1c levels
ESM Figure 2	Islet and mitochondrial cholesterol content

ESM Table 1: Human islet donor characteristics

Donor	Accession #	Gender	Age years	BMI kg/m^2	HbA1c mmol/mol (%)	Source	Used in Figure
1	SAMN08769836	F	56	33.5	33 (5.2)	Scharp / Lacy	1a, b, S1
2	SAMN08769831	F	51	20.1	37 (5.5)	U. Miami	1a, b, S1
3	SAMN08769819	F	45	30.5	49 (6.6)	SC-ICRC	1a, b
4	SAMN08769837	M	48	43.7	49 (6.6)	Scharp/ Lacy	1a, b
5	SAMN08612554	F	51	32.0	34 (5.3)	U. Miami	1a, c, S1
6	SAMN08612553	M	51	25.5	36 (5.4)	U. Pennsylvania	1a, c
7	SAMN18092805	M	56	21.6	32 (5.1)	SC-ICRC	1c
8	SAMN18528866	F	32	25.7	32 (5.1)	Scharp / Lacy	1c
9	SAMN18875704	M	37	28.4	38 (5.6)	Scharp / Lacy	1c
10	SAMN09228907	M	62	36.1	40 (5.8)	Scharp / Lacy	1a, S1
11	SAMN09370567	M	32	28.5	33 (5.2)	U. Wisconsin	1a, S1
12	SAMN09432880	M	51	35.7	36 (5.4)	SC-ICRC	1a, S1
13	SAMN09844981	M	56	29.4	60 (7.6)	SC-ICRC	1a
14	SAMN09862214	M	31	31.8	34 (5.3)	SC-ICRC	1a, S1
15	SAMN10372788	F	50	35.4	85 (9.9)	SC-ICRC	1a
16	SAMN11791244	M	47	36.1	39 (5.7)	SC-ICRC	1a, e, f, g, S1
17	SAMN12339206	M	45	32.9	36 (5.4)	SC-ICRC	1a, e, f, g, S1
18	SAMN12534700	M	29	26.0	43 (6.1)	U. Pennsylvania	1a, e, f, g, S1
19	SAMN40619409	M	63	34.1	32 (5.1)	Prodo Labs	1d, e, f, g, h, S1
20	SAMN40709610	F	55	28.6	39 (5.7)	U. Miami	1a, e, f, g, h, S1
21	SAMN41657868	M	38	25.5	36 (5.4)	U. Wisconsin	1a, e, f, g
22	SAMN42008301	M	36	30.0	31 (5)	U. Pennsylvania	1d, e, f, g, h
23	SAMN48072591	M	62	31.4	34 (5.3)	Prodo Labs	1g, h, S1
24	SAMN48430443	M	50	25.8	33 (5.2)	Prodo Labs	1g, h

Scharp-Lacy Research Institute (Prodo labs)
SC-ICRC: Southern California Islet Cell Resource Center (City of Hope)
HbA1c in red = confirmed T2D donor

ESM Table 2: Primary and secondary antibodies and buffers for protein detection by western blotting, immunohistochemistry, or immunofluorescence

Antibody	Vendor	Catalogue #	Species	RRID	Dilution for Western Blotting	Dilution for IHC / IF
**Primary Antibody**						
Insulin	Millipore Sigma	I2018	Mouse	AB_260137		1:2000
StAR	Cell Signaling Technology	8449S	Rabbit	AB_10889737	1:1000	1:100
Human StAR	LifeSpan Biosciences	C185729	Rabbit	AB_3740098	1:500	
StAR	Invitrogen	MA5-47013	Mouse	AB_2938085		1:100
HSP90	Cell Signaling Technology	4874	Rabbit	AB_2121214	1:1000	
ATP5a	Invitrogen	459240	Mouse	AB_2532234	1:1000	1:100
TOMM20	Abcam	ab289670	Rat	AB_2943038		1:100
**Secondary Antibody**						
anti-Rat AF488	Invitrogen	A11006	Goat	AB_2534074		1:250
anti-Mouse AF568	Invitrogen	A11004	Goat	AB_2534072		1:250
anti-Rabbit AF568	Invitrogen	A11011	Goat	AB_143157		1:250

**Buffers**	
**Purpose**	**Buffer**
Western blotting	Running Buffer: 0.3% (w/vol) Tris base (Sigma T1503), 1.44% (w/vol) glycine (Sigma G7126), pH 8.3, 0.1% (w/vol) SDS (Invitrogen 15553027)

Western blotting	Transfer Buffer: 0.3% (w/vol) Tris base, 1.44% (w/vol) glycine, pH 8.3, 0.1% (w/vol) SDS, 20% (vol/vol) Methanol (Sigma 179337)
IHC/IF	Blocking buffer: 1x PBS (Invitrogen AM9625), 0.2% (vol/vol) Triton X-100 (Thermo Fisher 85111), 0.01% (w/vol) sodium azide (Sigma S2002), 1% (w/vol) bovine serum albumin (Sigma A7888), 2% (vol/vol) normal goat serum (Gibco 16210064)
IHC/IF	Antibody incubation buffer: 1X PBS, 0.2% (vol/vol) Triton X-100, 0.01% (w/vol) sodium azide, 1% (w/vol) bovine serum albumin
IHC/IF	Wash buffer: 1X PBS, 0.2% (vol/vol) Triton X-100, 0.01% (w/vol) sodium azide
IHC/IF	Thioflavine S staining solution: 0.5% (w/vol) Thioflavine S (Sigma T1892) in water

ESM Table 3: TaqMan probes for gene expression analysis

Gene Name	Species	TaqMan Probe ID
Ppib (Cyclophilin)	Mouse	Mm00478295
PPIB (Cyclophilin)	Human	Hs00168719
Star (Stard1)	Mouse	Mm00441558
StAR (Stard1)	Human	Hs00986559
Stard3	Mouse	Mm00445524
Tspo	Mouse	Mm00437828

ESM Figure 1: Relationship of StAR mRNA expression in human islets with age, BMI and haemoglobin A1c levels:

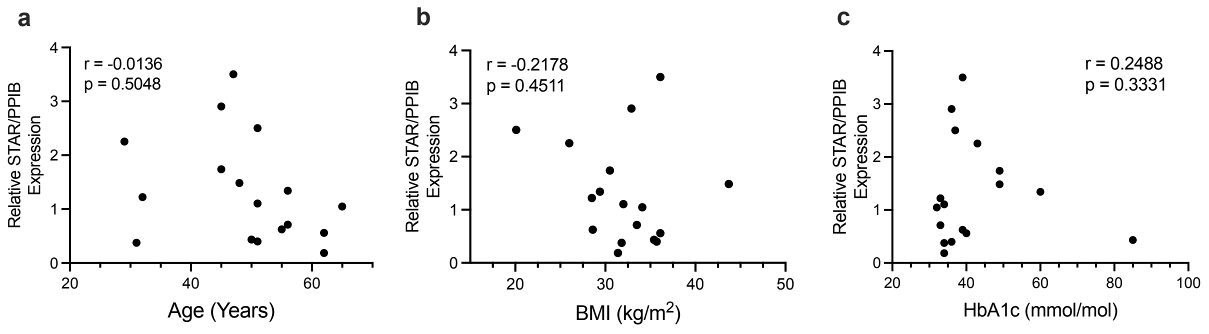

StAR mRNA expression in human islets does not correlate with (a) age, (b) BMI, and (c) haemoglobin A1c levels from individual donors without diabetes (n=13, see Supplemental Table 1).

ESM Figure 2: Islet and mitochondrial cholesterol content

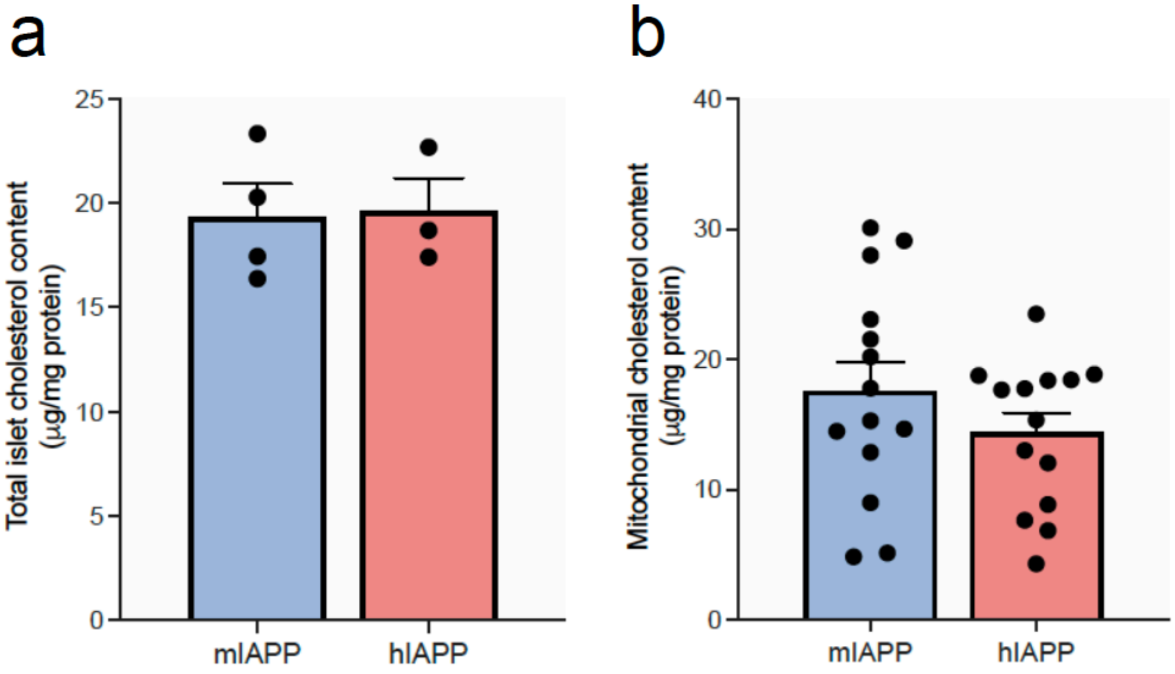

(a) Total islet cholesterol content relative to protein; n=3-4. (b) Mitochondrial cholesterol content relative to protein; n=14, in non-transgenic (mIAPP; blue bars) and RIP-hIAPP transgenic (red bars) islets.

Checklist for reporting human islet preparations used in research

Adapted from Hart NJ, Powers AC (2018) Progress, challenges, and suggestions for using human islets to understand islet biology and human diabetes. Diabetologia https://doi.org/10.1007/s00125-018-4772-2

**Islet preparation**	**1**	**2**	**3**	**4**	**5**	**6**	**7**	**8[a]**
**MANDATORY INFORMATION**								
Unique identifier	SAMN08769836	SAMN08769831	SAMN08769819	SAMN08769837	SAMN08612554	SAMN08612553	SAMN18092805	SAMN18528866
Donor age (years)	56	51	45	48	51	51	56	32
Donor sex (M/F)	F	F	F	M	F	M	M	F
Donor BMI (kg/m^2)	33.5	20.1	30.5	43.7	32	25.5	21.6	25.7
Donor HbA_{1c} or other measure of blood glucose control	33 mol/mol (5.2%)	37 mol/mol (5.5%)	49 mol/mol (6.6%)	49 mol/mol (6.6%)	34 mol/mol (5.3%)	36 mol/mol (5.4%)	32 mol/mol (5.1%)	32 mol/mol (5.1%)
Origin/source of islets[b]	IIDP	IIDP	IIDP	IIDP	IIDP	IIDP	IIDP	IIDP
Islet isolation centre	Sharp / Lacy	U. Miami	SC-ICRC	Sharp / Lacy	U. Miami	U. Pennsylvania	SC-ICRC	Sharp / Lacy
Donor history of diabetes? Please select yes/no from drop down list	No	No	Yes	Yes	No	No	No	No
**If Yes, complete the next two lines if this information is available**								
Diabetes duration (years)								
Glucose-lowering therapy at time of death[c]								

RECOMMENDED INFORMATION								
Donor cause of death	Head Trauma	CV / Stroke	Head Trauma	Anoxia	CV / Stroke	CV / Stroke	CV / Stroke	CV / Stroke
Warm ischaemia time (h)								
Cold ischaemia time (h)	11.3	14.9	0	7.5	14.5	15.5	5.7	8.5
Estimated purity (%)	90	90	80	90	90	90	90	85
Estimated viability (%)	95	95	97	95	90	93	96	95
Total culture time (h)[d]	84	75	92	61	94	45	36	91
Glucose-stimulated insulin secretion or other functional measurement[e]								
Handpicked to purity? Please select yes/no from drop down list	Yes	Yes	Yes	Yes	Yes	Yes	Yes	Yes
Additional notes								

[a]If you have used more than eight islet preparations, please complete additional forms as necessary
[b]For example, IIDP, ECIT, Alberta IsletCore

**Islet preparation**	**9**	**10**	**11**	**12**	**13**	**14**	**15**	**16**

MANDATORY INFORMATION								
Unique identifier	SAMN18875704	SAMN09228907	SAMN09370567	SAMN09432880	SAMN09844981	SAMN09862214	SAMN10372788	SAMN11791244
Donor age (years)	37	62	32	51	56	31	50	47
Donor sex (M/F)	M	M	M	M	M	M	F	M
Donor BMI (kg/m^2)	28.4	36.1	28.5	35.7	29.4	31.8	35.4	36.1
Donor HbA_{1c} or other measure of blood glucose control	38 mmol / mol (5.6%)	40 mmol / mol (5.8%)	33 mmol / mol (5.2%)	36 mmol / mol (5.4%)	60 mmol / mol (7.6%)	34 mmol / mol (5.3%)	85 mmol / mol (9.9%)	39 mmol / mol (5.7%)
Origin/source of islets[b]	IIDP	IIDP	IIDP	IIDP	IIDP	IIDP	IIDP	IIDP
Islet isolation centre	Scharp / Lacy	Scharp / Lacy	U. Wisconsin	SC-ICRC	SC-ICRC	SC-ICRC	SC-ICRC	SC-ICRC
Donor history of diabetes? Please select yes/no from drop down list	No	No	No	No	Yes	No	Yes	No
**If Yes, complete the next two lines if this information is available**								
Diabetes duration (years)								
Glucose-lowering therapy at time of death[c]								
**RECOMMENDED INFORMATION**								
Donor cause of death	Head Trauma	Anoxia	Head Trauma	CV / Stroke	CV / Stroke	Head Trauma	CV / Stroke	CV / Stroke
Warm ischaemia time (h)								

Cold ischaemia time (h)	11.5	10.7	11.5	6	6.5	5.5	8.5	6.5
Estimated purity (%)	90	90	95	80	75	80	80	95
Estimated viability (%)	95	95	91	96	93	95	96	95
Total culture time (h)[d]	65	92	42	102	44	45	67	389
Glucose-stimulated insulin secretion or other functional measurement[e]								
Handpicked to purity? Please select yes/no from drop down list	Yes	Yes	Yes	Yes	Yes	Yes	Yes	Yes
Additional notes								

**Islet preparation**	**17**	**18**	**19**	**20**	**21**	**22**	**23**	**24**
**MANDATORY INFORMATION**								
Unique identifier	SAMN12339206	SAMN12534700	SAMN40619409	SAMN40709610	SAMN41657868	SAMN42008301	SAMN48072591	SAMN48430443
Donor age (years)	45	29	63	55	38	36	62	50
Donor sex (M/F)	M	M	M	F	M	M	M	M
Donor BMI (kg/m^2)	32.9	26	34.1	28.6	25.5	30	31.4	25.8
Donor HbA_{1c} or other measure of blood glucose control	36 mmol / mol (5.4%)	43 mmol / mol (6.1%)	32 mmol / mol (5.1%)	39 mmol / mol (5.7%)	36 mmol / mol (5.4%)	31 mmol / mol (5%)	34 mmol / mol (5.3%)	33 mmol / mol (5.2%)
Origin/source of islets[b]	IIDP	IIDP	IIDP	IIDP	IIDP	IIDP	IIDP	IIDP
Islet isolation centre	SC-ICRC	U. Pennsylvania	Prodo Labs	U. Miami	U. Wisconsin	U. Pennsylvania	Prodo Labs	Prodo Labs
Donor history of diabetes? Please select yes/no from drop down list								
**If Yes, complete the next two lines if this information is available**								
Diabetes duration (years)								
Glucose-lowering therapy at time of death[c]								
**RECOMMENDED INFORMATION**								
Donor cause of death	CV / Stroke	Anoxia	Anoxia	CV / Stroke	Head Trauma	Anoxia	CV / Stroke	Head Trauma

Warm ischaemia time (h)								
Cold ischaemia time (h)	6.5	18.5	11.2	11.8	4	14	12	10
Estimated purity (%)	85	90	90	85	90	80	90	90
Estimated viability (%)	96	93	95	92	98	97	95	95
Total culture time (h)[d]	392	384	441	382	403	407	425	427
Glucose-stimulated insulin secretion or other functional measurement[e]								
Handpicked to purity? Please select yes/no from drop down list	Yes	Yes	Yes	Yes	Yes	Yes	Yes	Yes
Additional notes								

[c]Please specify the therapy/therapies
[d]Time of islet culture at the isolation centre, during shipment and at the receiving laboratory
[e]Please specify the test and the results
